# Alleviating the Effects of Electrolyzed Alkaline Water on Hyperuricemia in Mice

**DOI:** 10.3390/nu17101673

**Published:** 2025-05-14

**Authors:** Leihong Mao, Haiqin Zhao, Xiaoyan Tian, Yumei Qin, Guohua Li, Zihan Qin, Yuezhong Mao, Xiao Ye, Yanyun Cao, Shiyi Tian

**Affiliations:** 1 School of Food Science and Biotechnology, Zhejiang Gongshang University, Hangzhou 310018, China; 2 Shanghai Kangshi Food Technology Co., Ltd., Shanghai 201103, China; 3 Zhejiang-UK Joint Laboratory of Food Sensory Science, Zhejiang Gongshang University, Hangzhou 310018, China

**Keywords:** hyperuricemia, electrolyzed alkaline water, urate transporters, renal damage

## Abstract

**Objectives:** This study investigated the effects and mechanisms of electrolyzed alkaline water (EAW), a type of drinking water, on hyperuricemia (HUA) in mice. **Methods:** A hyperuricemia model was established by intraperitoneal injection of potassium oxonate and free access to a high-purine diet. EAW was provided ad libitum for 21 days. **Results:** The results showed that EAW had little impact on the levels of blood urea nitrogen, alanine aminotransferase, aspartate aminotransferase, albumin, or xanthine oxidase in mice (*p* > 0.05). Interestingly, EAW ingestion induced significant reductions in uric acid and creatinine levels (*p* < 0.05), along with increased urinary uric acid excretion (*p* < 0.05) and less renal pathological changes in mice. Additionally, EAW upregulated GLUT9 gene expression (*p* > 0.05) and downregulated URAT1 protein expression. **Conclusions:** In conclusion, this study demonstrates that EAW promotes uric acid excretion by downregulating URAT1 and GLUT9 protein expression, resulting in a significant reduction in uric acid levels.

## 1. Introduction

Hyperuricemia, which refers to elevated levels of uric acid in the blood, is a metabolic disorder caused by impaired purine metabolism, which can lead to gout, diabetes, chronic kidney disease, and other related disorders. The condition arises from an imbalance between uric acid production and excretion. Several factors, such as diet, genetics, and environmental factors, contribute to this condition. Specifically, changes in lifestyle and diet—such as increased consumption of meat and processed foods—has led to a significant rise by approximately 20% in the global incidence of hyperuricemia [1]. For example, the prevalence is estimated at 16.4% in China, with a trend of younger age groups being affected [2], while the prevalence reaches 20% in adults in the U.S. [3] and 26.6% in Korea [4]. Obviously, hyperuricemia has become a major public health issue worldwide. However, in the development of the asymptomatic phase, hyperuricemia often shows no clinical signs, and treatment with uric acid-lowering medications is not recommended for asymptomatic individuals [5]. Therefore, identifying safe and effective treatments for preventing hyperuricemia is essential as an alternative to medical treatment.

Several strategies to prevent hyperuricemia have been suggested. For example, a regular routine and good diet can go some way toward preventing it, including the consumption of low-purine foods (coarse grains, vegetables, fruits, etc.) and the establishment of a good lifestyle (doing exercise, having fewer or even no late nights, consuming less or even no alcohol and coffee [6], etc.). It has also been reported that the consumption of functional foods may be considered a pharmaceutical alternative for the prevention or treatment of hyperuricemia [7]. For example, plant-based functional foods contain a wide range of naturally beneficial phytochemicals. However, the applicability of the bioactive ingredients in plant-based functional foods to humans is unclear, and excessive intake may increase the risk of kidney stones.

Electrolyzed alkaline water (EAW, pH 9.0 ± 0.5), which is produced through electrolysis, has a higher pH value than that of regular drinking water (neutral water with a pH value of about 7) and is rich in minerals [8,9]. EAW has been reported to show physiological efficacies. For example, EAW was able to scavenge active oxygen species and therefore protects DNA from oxidative damage [8]. EAW has been proven to be beneficial in the treatment of patients with reflux disease [10]. In addition, EAW may be considered a primary safe strategy for the treatment of metabolic acidosis caused by renal failure or dialysis or urinary diversion [11].

EAW is characterized by an elevated pH and abundance of alkaline minerals such as calcium, magnesium, and potassium, which may affect renal urate treatment through a variety of mechanisms. Elevated pH in the systemic circulation and urine may increase uric acid excretion [12]. Drinking mineral water will improve the relative saturation of uric acid in the body [13]. Furthermore, alkaline minerals may modulate the expression and activity of urate transporters in the proximal tubules, such as URAT1, GLUT9, and OAT1/3. For instance, an alkaline urinary environment can suppress the URAT1-mediated reabsorption of urate, while the upregulation of ABCG2 and OAT1 may promote urate secretion [14]. Additionally, the antioxidant properties often associated with EAW—due to dissolved molecular hydrogen and ORP (oxidation-reduction potential)—may attenuate oxidative stress in renal tissue, which is known to influence transporter regulation through redox-sensitive signaling pathways [15]. Together, these physicochemical and molecular effects suggest that EAW may beneficially modulate renal urate clearance, potentially lowering serum uric acid levels. However, there have been no reports on whether EAW has a protective role in hyperuricemia.

Therefore, this study aims to investigate the effects of ad libitum consumption of electrolyzed alkaline water (EAW) in a hyperuricemia mouse model, examining its impact from molecular and genetic perspectives and providing data to support the development of safe and effective strategies for hyperuricemia prevention.

## 2. Materials and Methods

### 2.1. Materials

Commercial drinking water (pH 6.5), as well as EAW drinking water (pH 9.0 ± 0.5), was supplied by Shanghai Kangshi Food Technology Co., Ltd. (Shanghai, China). Potassium oxonate and carboxymethylcellulose sodium were purchased from Sigma-Aldrich (St. Louis, MO, USA). Paraformaldehyde (4%, *w*/*v*) was purchased from Biosharp Life Science (Hefei, China). High-purine feed and normal feed were provided by Xietong Shengwu Co. (Nanjing, China). GAPDH, URAT1 Antibody was purchased from Affinity Biosciences (Cincinnati, OH, USA), while GLUT9 Antibody was purchased from Abcam (Cambridge, UK).

### 2.2. Experimental Animals

Animal experiments were performed in accordance with the regulations outlined by Dracon Laboratory Animal Research Centre (Hangzhou, China). All experimental protocols were approved by The Lab of Animal Experimental Ethical Inspection of Dr. Can Biotechnology (Hangzhou, China) Co., Ltd. (Reference Number: DRK-20240912010). Healthy male C57BL/6 mice (32) with a body weight of (23 ± 1) g were purchased from Hangzhou Hangs Biotechnology Co., Ltd. (Hangzhou, China). The animals were acclimated for 7 days under controlled conditions (24 ± 2 °C, 55–65% relative humidity, and a 12/12 h light/dark cycle), with free access to food and water. After acclimatization, the mice were randomly assigned to treatment groups.

### 2.3. Establishment and Grouping of Hyperuricemia Models

Based on feeding conditions, all animals were randomly assigned to four groups (8 mice in each group): control, model, EAW, and allopurinol. Hyperuricemia was induced in the model, EAW, and allopurinol groups using a high-purine diet (0.2% adenine + 20% yeast powder, provided ad libitum) combined with daily intraperitoneal injections of potassium oxonate (200 mg/kg) for 21 days. The control group received a standard diet, had free access to bottled water, and were injected with 0.5% carboxymethylcellulose sodium (CMC-Na) solution (0.1 mL/10 g body weight). The allopurinol group was fed a high-purine diet, given free access to control water, injected with potassium oxonate dissolved in 0.5% CMC-Na (200 mg/kg, 0.1 mL/10 g), and administered allopurinol solution orally (40 mg/kg, 0.1 mL/10 g). The model group received the high-purine diet and control water, along with intraperitoneal injections of potassium oxonate dissolved in 0.5% CMC-Na. The EAW group was given the high-purine diet and EAW, along with the same oxonate injections as the model group. Body weights were recorded daily throughout the experiment. Euthanasia was carried out by cervical dislocation under deep anesthesia, following the administration of 4% chloral hydrate at a dose of 0.1 mL per 10 g of body weight.

### 2.4. Measurement of Serum Levels of Uric Acid (UA), Creatinine (CRE), Blood Urea Nitrogen (BUN), Alanine Aminotransferase (ALT), Aspartate Aminotransferase (AST) and Albumin

At the end of the modeling phase and after the intervention, blood samples were collected from the retro-orbital sinus of mice in each group. Blood was collected in a 1.5 mL centrifuge tube and left to stand at room temperature for 30 min, followed by centrifugation at 4500× *g* for 30 min at low temperature. All the parameters, including UA, CRE, BUN, ALT, AST, and albumin, were measured using commercially available colorimetric assay kits from Nanjing Jiancheng Bioengineering Institute Co., Ltd. (Nanjing, China). The specific kit catalog numbers were as follows: UA (C012-2-1), CRE (C011-2-1), BUN (C013-2-1), ALT (C009-2-1), AST (C010-2-1), and albumin (A028-2-1). All assays were performed manually in accordance with the manufacturer’s instructions.

### 2.5. Liver and Serum Xanthine Oxidase (XOD) Activity Measurements

At the end of modeling and intervention, about 100 mg of mouse liver tissue was weight, 1 mL of saline was added, and it was homogenized at 4 °C or in an ice bath and centrifuged at 4 °C and 3000× *g* for 10 min. Liver supernatant and serum were taken to determine the activity of XOD according to the instructions of the assay kit (Nanjing Jiancheng Bioengineering Institute Co., Ltd., Nanjing, China).

### 2.6. Metabolic Cage Experiments

Metabolic cage (Suzhou New District Fengqiao Purification Equipment Co., Ltd., Suzhou, China) experiments were conducted at the end of the modeling phase and after the intervention. Twenty-four-hour urine samples were collected to measure urine volume and uric acid (UA) levels, the latter determined using standard biochemical methods.

### 2.7. Pathological Examination

Following the completion of metabolic cage experiments at the end of the modeling and intervention phases, mice were euthanized via spinal dislocation. The left kidney was collected, fixed in 4% paraformaldehyde, and processed for paraffin embedding. Tissue sections were prepared and stained with hematoxylin and eosin (HE). Histological changes in the kidneys of each group were examined under a light microscope (Nikon Corporation, Tokyo, Japan). Two independent, blinded experts with relevant experience evaluated the histopathology slides.

### 2.8. mRNA Expression of Genes Related to Uric Acid Metabolism

About 50 mg of right kidney tissue was placed in a sterile EP tube containing 1 mL of Trizol lysate and homogenized with an electric homogenizer. After homogenization, the supernatant was added with chloroform and other reagents, total RNA was extracted, and the concentration was determined. Then, cDNA was synthesized by reverse transcription according to the instructions of the kit (Vazyme, Nanjing, China), real-time fluorescence PCR (Bio-Rad Laboratories, Hercules, CA, USA) was carried out by the fluorescent dye infiltration method, and the relative expression amount of the genes was calculated with the 2^−ΔΔCt^ method [16].

### 2.9. Uric Acid Metabolism-Related Protein Expression

About 20 mg of the right kidney was weighed into an EP tube, and 200 μL of RIPA lysis solution (containing PMSF and phosphatase inhibitor) was added. This was then centrifuged at 13,400× *g* and 4 °C for 5 min, and the tissue was well broken up by using a motorized milling instrument on ice, followed by an ice bath for 30 min to sufficiently lysis the tissue. Then, this was centrifuged at 13,400× *g* and 4 °C for 5 min to obtain the supernatant, which was then quantified by using a BCA Protein assay kit (P0012, Beyotime Biotech Inc., Shanghai, China). The supernatant was extracted, and the total protein was quantified by the BCA protein quantification method. An appropriate amount of protein sample buffer was added to the EP tube with protein supernatant, this was heated at 100 °C for 5 min, and the proteins were denatured well and stored at −80 °C. After protein separation by SDS–polyacrylamide gel electrophoresis (SDS-PAGE), they were transferred to a PVDF membrane, and the primary antibody was incubated at 4 °C overnight after cutting the target bands. On the following day, the strips were incubated with horseradish peroxidase-coupled secondary antibody in an appropriate washing solution for 10 min × 4 times at room temperature for 1 h. Subsequently, the strips were washed with washing solution for 10 min × 3 times; the washed strips were submerged in the configured chemical solution for luminescence development, and the protein strips were imaged by the Chemi Doc MP Imaging System and quantified in gray using Image J 1.53q software (NIH, Bethesda, MD, USA). The protein bands were imaged by Chemi Doc MP Imaging System and quantified by Image J software.

### 2.10. Statistical Analysis

The data obtained from the experiment were statistically analyzed using Graphpad prism 8.0.2 (GraphPad Software Inc., La Jolla, CA, USA) and SPSS Statistics 26.0 (SPSS Inc., Chicago, IL, USA). Significant differences were analyzed by one-way ANOVA (Tukey’s test). The results of the experiment were exhibited as the mean ± standard deviation ($\bar{X}$ ± SD), *p* < 0.05, which were used to assess statistical significance.

## 3. Results

### 3.1. Weight Changes

The changes in the body weights of each group are shown in Table 1, and the body weights were recorded from the beginning of the experiment to the end of the experiment once a day. There was no statistically significant difference in body weight between groups after the feeding acclimatization of animals in each group. Before modeling, there was no difference in body weight between the groups. At the end of modeling, the control group gained 8% of their body weight; the allopurinol group lost about 11% of their body weight; the model group lost about 25% of their body weight; and the EAW group lost about 23% of their body weight. Hyperuricemia modeling had a significant effect on body weight (*p* < 0.05), and daily intraperitoneal injections of potassium oxonate administered to the mice may have led to abdominal pain and a reduction in standing while feeding, which in turn led to a significant decrease in body weight. From the body weight data, it can be concluded that alkaline water did not have a significant mitigating effect on weight loss.

### 3.2. Urine and Water Intake

The results of water intake in the mice are shown in Table 2. The results showed that modeling increased the water intake of mice. Compared to the first week, the water intake of mice in the control, allopurinol, modeling, and EAW groups increased by 9.52%, 20.7%, 46.96%, and 27.95% in the second week and 4%, 12.76%, 49.82%, and 23.06% in the third week, respectively. Compared with the control group, the water intake of mice in the allopurinol group, the model group, and the EAW group was higher than that of the control group with the increase in feeding time (the first, second, and third weeks).

**Figure 1:**
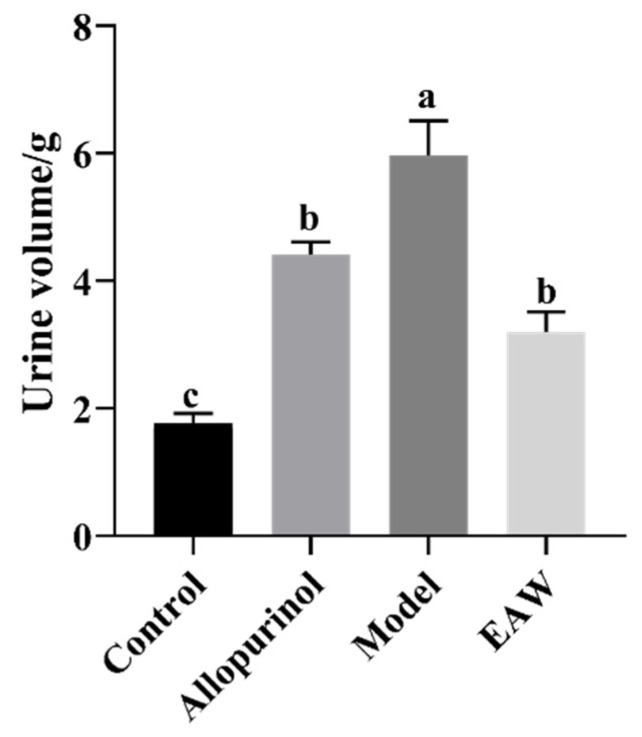
Urine volume of mice in each group. Different letters (a–c) indicate significant differences (*p* < 0.05).

The results for the urine volume of the mice are shown in Figure 1. The analysis of the urine volume of the mice showed that the control group mice had the lowest urine volume and the model group mice had a significantly higher urine volume than the other groups. The urine volume of the allopurinol, model, and EAW groups was higher than that of the control group; however, no significant difference was presented between the allopurinol and EAW groups.

### 3.3. Analysis of Physiological Indexes Related to UA Metabolism in Mice of Each Group

**Figure 2:**
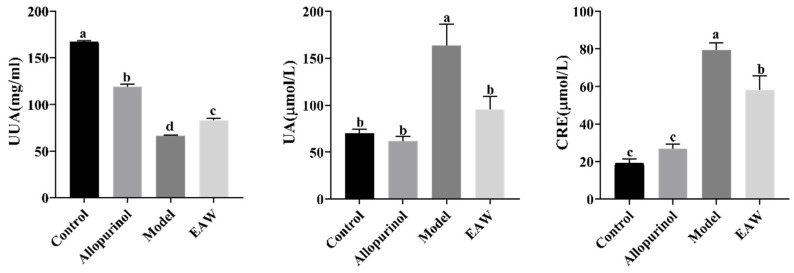
Urinary uric acid (UUA), serum uric acid (UA), and serum creatinine (CRE) levels in mice of each group. Different letters (a–d) indicate significant differences (*p* < 0.05).

If the body produces too much uric acid for excretion or the excretion mechanism of uric acid is degraded, too much uric acid will be retained in the body, resulting in the acidification of body fluids, which will cause gout in the long run. The creatinine level is mainly determined by the glomerular filtration capacity: with a decrease in the filtration capacity, the creatinine level will increase [17]. The results for urinary and serum UA and serum CRE levels in the mice are shown in Figure 2. There was a significant difference between the serum uric acid levels of the mice in the control group and the model group, indicating successful modeling. Compared with the control group, the serum UA and CRE levels were elevated in the model group of mice, and the difference was statistically significant (*p* < 0.05). Compared with the model group, the levels of UA and CRE were reduced to a certain extent in all groups, including the levels in the EAW group and the allopurinol group, and the difference was statistically significant (*p* < 0.05).

In this study, the uric acid levels in the urine of mice were examined. The results showed that compared with the control group, the urinary uric acid (UUA) level in mice was reduced, and the difference was statistically significant (*p* < 0.05). Compared with the model group, the UUA level was increased to some extent in all groups. The UUA level was increased in the EAW group and the allopurinol group, and the difference was statistically significant (*p* < 0.05), with the results shown in Figure 2.

**Figure 3:**
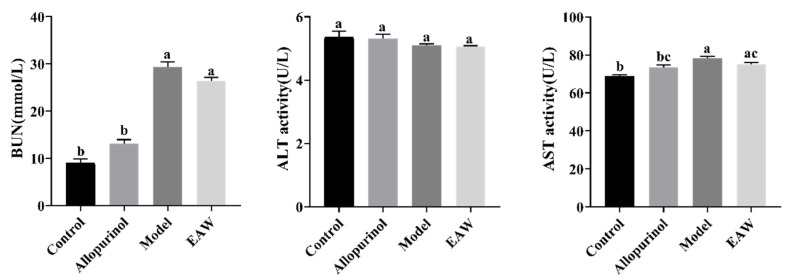
Serum blood urea nitrogen (BUN), alanine aminotransferase (ALT), and aminotransferase (AST) levels of mice in each group. Different letters (a–c) indicate significant differences (*p* < 0.05).

BUN levels are another important clinical indicator of glomerular filtration function. Elevated BUN is common in renal insufficiency but is usually seen when the glomerular filtration rate (GFR) is reduced by up to 50%, which was not observed in the present study (Figure 3). ALT is a sensitive marker of acute hepatocellular damage [18], while serum AST activity can be markedly elevated in cases of acute viral hepatitis. In the present study, no significant difference was observed in BUN, ALT, and AST levels between the EAW and model groups. However, a slight increase in AST levels was observed in the model group compared to the control group, indicating that hyperuricemia would have a detrimental effect on the liver (Figure 3).

**Figure 4:**
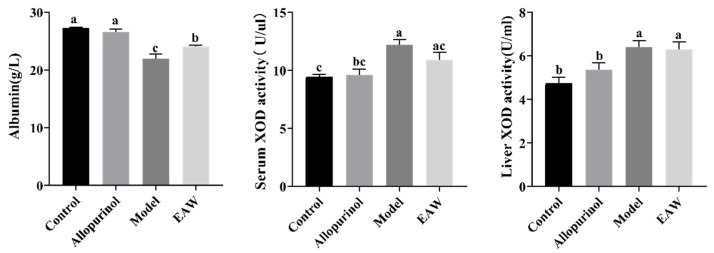
Serum albumin, serum XOD, and liver XOD levels in each group of mice. Different letters (a–c) indicate significant differences (*p* < 0.05).

The albumin level indicates the health of an organism’s kidneys, and a decrease in albumin level is an important indicator of impaired kidney function. As shown in Figure 4, the albumin level was significantly lower in the model group compared with the control group (*p* < 0.05), indicating that the renal function of the mice in the model group was impaired. The albumin level in the EAW group was lower than that in the control group but higher compared with the model group (*p* < 0.05), and the treatment in the EAW group elevated the serum albumin level of the mice. This indicates that EAW plays a mitigating role in kidney injury caused by hyperuric acid. XOD exists in various organisms and catalyzes the formation of UA from purine substrates in vivo. The higher the value of XOD, the greater the ability of the animal to generate UA. As shown in Figure 4, the serum and liver XOD activities of the model group were significantly higher than those of the control group. Although a reduction in XOD activity was observed with the EAW treatment (especially serum XOD), this change did not reach statistical significance. Therefore, attributing the urate-lowering effect of EAW primarily to suppressed uric acid production via XOD inhibition is not strongly supported by the data. Instead, given the significant downregulation of *URAT1* and *GLUT9* protein levels—key transporters involved in renal urate reabsorption—it is more plausible that EAW facilitates uric acid excretion rather than inhibiting its production.

### 3.4. Histopathologic Findings in the Kidneys

**Figure 5:**
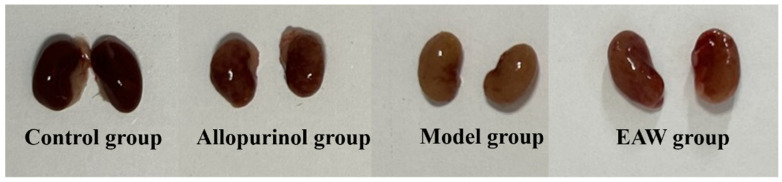
Image of the kidneys of mice in each group at the end of modeling.

Kidneys from each group are shown in Figure 5. The kidneys of the mice in the control group showed a dark red color, the kidneys in the allopurinol group showed a few yellowish spots, the kidneys in the model group showed a yellowish color, and the kidneys in the EAW group showed many yellowish spots, but fewer compared with the model group.

**Figure 6:**
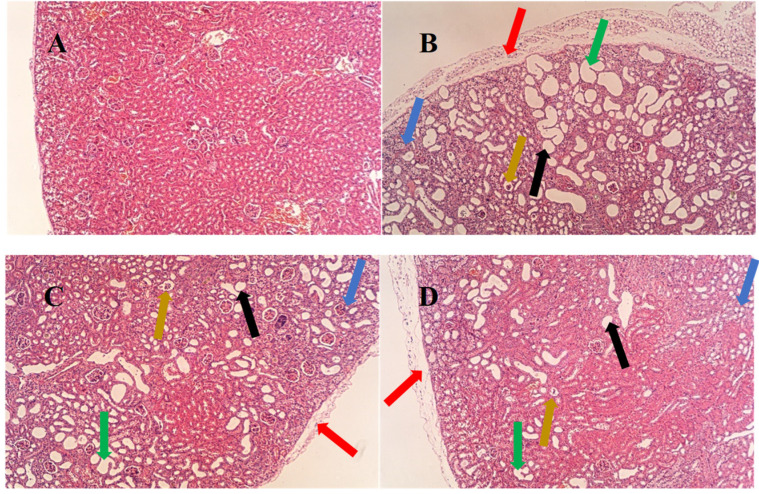
Histopathologic changes in the kidneys of mice in each group (HE, 40×). Note: (**A**–**D**) are the control, model, EAW, and allopurinol groups, respectively. The blue arrows indicate inflammatory infiltration; the red arrows indicate thickening of the renal peritoneum; the green arrows indicate the disappearance of glomeruli; the yellow arrows indicate glomerular atrophy; and the black arrows indicate tubular dilatation.

The histopathological results for the mouse kidney are shown in Figure 6. The kidneys in the control group had a normal histological structure: the tubular epithelial cells had no edema, were neatly arranged, and the structure was clear and intact. In the model group, compared with the normal group, some of the cells appeared to have vacuole-like changes [19,20], renal interstitial inflammatory cell infiltration [16], tubular dilatation [19], or renal tubular atrophy, or they even disappeared [19,20], and the renal tunic was thickened and edematous [16,21]. In contrast to the model group, the vacuole-like changes in the allopurinol group and the EAW group were relatively minor, the renal interstitial inflammatory cell infiltration was reduced by different degrees, the renal tubular atrophy was reduced, and the hyperuricemia-induced renal lesions were alleviated.

### 3.5. Renal mRNA Expression of UA Transporter Genes

**Figure 7:**
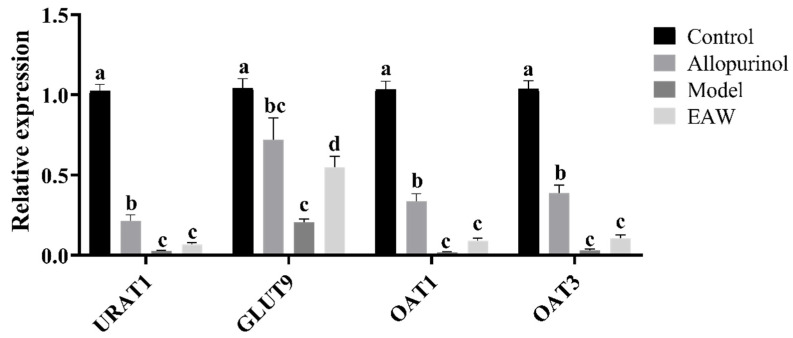
Relative mRNA expression in kidney tissues of mice in each group. Different letters (a–d) indicate significant differences (*p* < 0.05).

Uric acid transporter protein 1 (URAT1) is expressed mainly in the apical brush border membrane of renal proximal tubular epithelial cells and transports urate from the lumen of the proximal renal tubule to the tubular epithelial cells. OAT1 and OAT3 (Organic Anion Transporters) are located in the basolateral membranes of the renal tubular epithelial cells and mediate the secretion of urate into the lumen of the renal tubule in order to reduce UA levels. Glucose transporter protein 9 (GLUT9) is expressed in renal proximal tubules and has the ability to transport and efflux urate and reabsorb urate from the lumen of renal tubules. The relative expression of mRNA in the renal tissues of the mice in each group is shown in Figure 7.

The results showed that the expression of renal URAT1, OAT1, and OAT3 mRNA was significantly lower in the model group compared with the control group, indicating that the modeling of hyperuricemia was successful, and hyperuricemia led to renal injury, which in turn led to a significant decrease in the expression of the relevant genes. Compared with the model group, the expression of URAT1, OAT1, and OAT3 genes was elevated in the EAW group compared with the model group but did not show statistical differences; the genes URAT1, OAT1, and OAT3 were elevated in the allopurinol group.

Compared with the control group, the expression of renal GLUT9 mRNA in the model group was significantly reduced (*p* < 0.05). Compared with the model group, the expressions of GLUT9 mRNA in the mice in the EAW group were all significantly higher, indicating that EAW could reduce blood UA levels and alleviate kidney injury by reducing UA reabsorption and promoting UA excretion.

### 3.6. Renal Uric Acid Transporter Protein Expression

**Figure 8:**
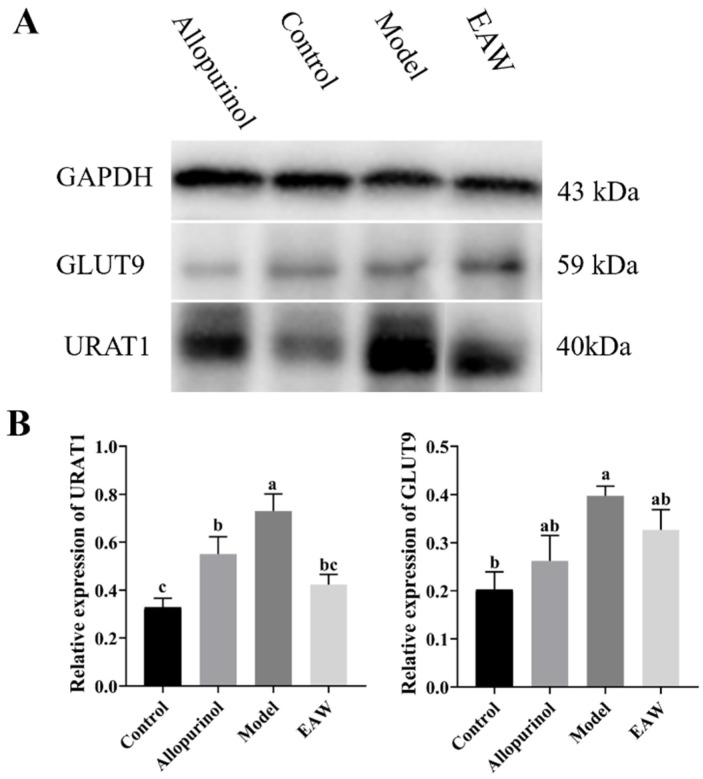
(**A**,**B**) Relative expression of URAT1 and GLUT9 proteins in kidney tissues of mice in each group. Different letters (a–c) indicate significant differences (*p* < 0.05).

Urate transporter proteins are responsible for UA excretion in an organism, and the effect of EAW on UA excretion can be determined by examining the changes in the relative expression of urate transporter proteins. URAT1 is expressed on the surface of proximal renal tubular epithelial cells and participates in the transport of UA from the kidneys to the bloodstream, and it is an important mechanism for regulating the level of UA in the blood. GLUT9, as a member of the glucose transporter family, can transport glucose to accelerate UA reabsorption. As shown in Figure 8, the protein expression levels of URAT1 and GLUT9 in the model group were significantly higher compared with the control group (*p* < 0.05), and the protein expression levels of URAT1 and GLUT9 in the EAW group were reduced compared with the model group with significant differences (*p* < 0.05).

## 4. Discussion

Hyperuricemia is a metabolic disorder in which the level of uric acid in the blood is too high (above 420 μmol/L in men and above 360 µmol/L in women) [22]. Elevated serum UA levels above the saturation point for urate (MSU) crystal formation cause gout [23].

In the present study, EAW was determined to significantly alleviate hyperuricemia by inducing hyperuricemia through the combined use of increased UA sources and the inhibition of uricase function. In the present study, after the last administration of the drug, the UA levels in the serum and urine of mice in each group were detected, and it was found that the UA levels in the serum of the model group were significantly higher than those of the control group (*p* < 0.05), and the UA levels in urine of the model group were significantly lower than those of the control group (*p* < 0.05). This indicated that the modeling of the mice, carried out by allowing the mice to freely consume high-purine feed in combination with an intraperitoneal injection of potassium oxalacetate, was successful. Compared with the model group, the serum UA level and Cre were significantly lower in the EAW and allopurinol groups (*p* < 0.05), and the urine UA level was significantly higher (*p* < 0.05). In vivo Cre and BUN levels are often used as indicators of renal injury [24]. According to Hoffmann et al. (2010), renal damage can impair the clearance of urea and creatinine (CRE), leading to elevated serum blood urea nitrogen (BUN) and CRE levels [25]. In the present study, EAW administration significantly reduced serum CRE levels and showed a non-significant trend toward reducing BUN levels. Similar results were reported by Guo et al. (2023) [26], who demonstrated a protective effect of α-viniferin against hyperuricemia-induced kidney injury in mice. AST and ALT are indicators for evaluating liver function [27]. No significant difference in ALT and AST levels was seen between the EAW and model groups. However, the slight increase in AST levels in the model group compared to the control group suggests that hyperuricemia may contribute to liver damage, consistent with findings by Liu et al. (2023) [28]. Albumin levels will indicate the health of the kidneys [29]. The albumin level in the EAW and allopurinol groups was significantly higher than that in the model group (*p* < 0.05), and EAW had a protective effect against renal injury in the hyperuric acid model mice. XOD exists in a variety of organisms and catalyzes the formation of purine substrates in vivo with UA [30]. The decrease in XOD activity in the EAW group was not significant (*p* > 0.05), indicating that EAW may not prevent UA production by inhibiting XOD activity. Meanwhile, through the HE staining of the kidneys of the mice in each group, it was found that the renal tissues of the mice in each group, except the control group, had different degrees of alteration, which showed that EAW had a better protective effect on the kidneys.

OAT1 and OAT3 are located in the basolateral region of proximal tubular epithelial cells and are key proteins involved in uric acid secretion [31]. However, in our study, we found that there was no significant elevation of OAT1 and OAT3 mRNA expression in the EAW group compared to the model group (*p* > 0.05). URAT1 and GLUT9 are two key members of the urate transporter protein family responsible for reabsorption of urate from the renal lumen to proximal tubular cells [32]. Interestingly, while the mRNA expression levels of URAT1 and GLUT9 remained the same, their corresponding protein levels were significantly reduced. This difference suggests underlying post-transcriptional regulatory mechanisms [33], such as altered mRNA stability, translational repression, or increased protein degradation [34]. Differences in protein half-lives may also contribute to this difference [35]. Gene polymorphisms may also lead to misalignment between mRNA and protein expression trends [36]. These findings highlight that transcriptional data alone may not be fully representative of functional protein expression. Therefore, the observed regulation of EAW on uric acid treatment may be driven by post-translational or translational regulation. In the present study, the expression of URAT1 and GLUT9 proteins in the renal tissues of mice in the model group was obviously increased, and the expression of URAT1 and GLUT9 proteins was significantly reduced after treatment with EAW and allopurinol compared with that of the model group (*p* < 0.05), suggesting that the EAW may reduce the effects of UA in vivo by preventing UA reabsorption and consequently increasing UA excretion.

## 5. Conclusions

In this study, EAW appears to have an alleviating effect on hyperuricemia in mice, potentially by lowering uric acid levels and mitigating kidney damage. EAW promoted UA excretion by downregulating URAT1 and GLUT9 protein expression. These effects could offer a promising natural remedy for managing hyperuricemia and its associated complications. Nonetheless, future studies should elucidate the underlying mechanisms, including the potential modulation of urate transporter expression via post-transcriptional regulation and the inflammatory responses induced by EAW in hyperuricemic mice. Additionally, more extensive research, particularly clinical trials in humans, is required to confirm its long-term efficacy and safety.

## Figures and Tables

**Table 1:** Changes in body weight of mice in each group.

Groups	n	Starting Weight (g)	Terminal Weight (g)	Weight Gain (g)
Control group	8	25.09 ± 0.80	26.7 ± 0.72 ^Δ^	1.8 ± 1.02 ^Δ^
Allopurinol group	8	25.40 ± 0.74	22.9 ± 0.88 *^,Δ^	−2.5 ± 1.23 *^,Δ^
Model group	8	25.14 ± 0.43	19.14 ± 1.06 *	−6.2 ± 1.31 *
EAW group	8	25.61 ± 0.59	19.98 ± 0.46 *^,Δ^	−5.1 ± 0.39 *^,Δ^

* *p* < 0.05 compared with control group; ^Δ^  *p* < 0.05 compared with model group.

**Table 2:** Changes in water intake of mice in each group.

Groups	n	The First Week (g)	The Second Week (g)	The Third Week (g)
Control group	8	15.94 ± 2.74 ^Δ^	17.46 ± 2.95 ^Δ^	16.58 ± 3.35 ^Δ^
Allopurinol group	8	19.45 ± 3.44 *	23.47 ± 1.48 *^,Δ^	21.93 ± 0.66 *^,Δ^
Model group	8	20.15 ± 1.50 *	29.61 ± 0.15 *	30.18 ± 2.99 *
EAW group	8	20.21 ± 1.54 *	25.85 ± 1.45 *^,Δ^	24.87 ± 0.92 *^,Δ^

* *p* < 0.05 compared with control group; ^Δ^  *p* < 0.05 compared with model group.

## Data Availability

The original contributions presented in this study are included in the article. Further inquiries can be directed to the corresponding authors.
